# Acoustic scattering and mode interaction in bifurcated circular waveguide structures with reacting liners and discontinuities

**DOI:** 10.1371/journal.pone.0321050

**Published:** 2025-05-23

**Authors:** Naif Alkuhayli, Aqsa Yaseen, Rab Nawaz, Hani Alahmadi

**Affiliations:** 1 Department of Mathematics, College of Science, Jouf University, Sakaka, Saudi Arabia; 2 Department of Mathematics, COMSATS University Islamabad, Islamabad, Pakistan; 3 Center for Applied Mathematics and Bioinformatics (CAMB), Gulf University for Science and Technology, Hawally, Kuwait; Beni-Suef University, EGYPT

## Abstract

Acoustic scattering and nonlinear dispersion phenomena play a crucial role in the design and optimization of waveguide systems for noise control in engineering applications such as HVAC systems, aircraft engines, and industrial gas turbines. In this study, we investigate the scattering characteristics of a bifurcated circular cylindrical waveguide with a particular focus on the effects of reactive acoustic liners and step-discontinuities. Unlike prior studies that typically analyze either acoustic liners or step discontinuities in isolation, this work integrates both mechanisms within a unified framework, providing new insights into their combined influence on wave propagation. The impedance conditions at the fluid-liner interface are formulated to evaluate scattering under different configurations, while the mode-matching (MM) technique is employed to determine eigenfunction expansions and solve the governing equations. The accuracy of the solution is validated through power conservation and matching condition reconstruction, ensuring the robustness of the methodology. The findings reveal that reacting liners effectively attenuate fluid-borne modes by preventing transmission up to a critical frequency where secondary modes become dominant, whereas step-discontinuities exhibit the opposite trend. The study further demonstrates that scattering behavior can be optimized by adjusting the radii of circular waveguide regions, leading to enhanced performance under liner conditions compared to step-discontinuities. Additionally, varying liner sizes or discontinuity heights significantly affects energy flux reflection and transmission, with higher frequencies amplifying scattering effects. These results provide a comprehensive framework for designing advanced noise control solutions in waveguide systems and offer valuable guidelines for practical engineering applications.

## Introduction

Acoustic liners have applications in noise control device for heating, ventilation and air conditioning (HVAC) systems, aircraft engines and industrial gas turbines [1, 2]. Recent advances in the design and optimization of structural systems have underlined the need for effective control methods to improve performance and stability. For example, Feng *et al*. [3] investigated vibration control and resilience in tensegrity structures utilizing a fuzzy dynamic sliding mode control method, exhibiting considerable gains in structural vibration management with sophisticated control strategies in complicated circumstances. Similarly, Nguyen-Thai *et al*. [4] developed an optimized design approach for passive viscous damping control in multi-story buildings using fluid viscous dampers (FVDs) and viscoelastic dampers (VWDs), which effectively reduced vibration levels and improved structural resilience under dynamic loading conditions. These investigations highlight the possibility of tailored control strategies to reduce undesirable dynamic reactions, a concept that also applies to acoustic waveguides, where managing scattering and dispersion processes is critical for maximizing acoustic performance. While considering the acoustic liners, the bulk-reacting liners and locally reacting liners are considered to be two crucial types. The bulk-reacting liners contain porous material are capable of sound dissipation in wider frequency band. Nevertheless, such devices are suitable on higher frequencies only, and are unable to attenuate noise in low frequencies regime. On the other hand, the locally reacting liners are composed of perforated plate backed by honey comb layers that provide internal partitions and are usually attached to the inner walls of nacelle to attenuate aircraft engine noise. Their main advantage is the ability to resist tough conditions and mechanical robustness, but can attenuate noise in narrow frequency band only. However, to enhance the efficiency of acoustic liners in targeted range of frequencies various strategies are used, for instance one may choose a material design having impedance to get the optimal range [5–7] and/or may consider geometric variations [8–11].

The pipes or ducts are essential part of the many engineering designs, for instance, as in HVAC, automobiles, aircraft jet and turbofan engines. Their main objective is to distribute air, heat and ventilation, but they are efficient carrier of unwanted noise as well. The noise generated by particular source propagates through these air ducts and produce noise at a removed distance. To minimize the unwanted duct noise different structural designs and various material properties are used. Rawlins [12] considered parallel partitioning along with acoustically absorbent lining in an unflanged cylindrical duct to mitigate the ducted fan noise. He solved the governing boundary value problem by using the modified Wiener-Hopf (WH) technique. Demir and Cinar [13] analyzed sound scattering and absorption from coaxial circular waveguide in which the inner tube comprises uniform gas flow and outer tube contains discontinuous wall impedances. Accordingly, the acoustic propagation and attenuation through waveguides having bifurcation and trifurcation are discussed in [14–22].

In the past few years, acoustical liners to minimize the noise produced by aircraft engines by damping the inlet/outlet acoustic modes have been extensively applied. Typically, these are locally reacting liners formed by honeycomb layers separated to airway by porous screen. Bi *et al*. [23] considered a circular chamber encompassing reacting liners connected to extended rigid inlet/out. The impedance of lining varies circumferentially and remains invariant in axial direction. They found solution of their problem by using improved multimodal technique. The technique relies on the projection of acoustic pressure over rigid duct modes, whereas, the information of impedance boundary is formulated by introducing additional priori solutions. In literature, acoustic liners are treated as impedance type of boundary condition with uniform or variable impedance, for instance see [24–26].

Despite the progress in acoustic liner design and step-discontinuity analysis, several key questions remain unanswered: How do reacting liners and step-discontinuities interact in a bifurcated waveguide? What are the optimal liner configurations and geometric parameters for minimizing transmission losses? Can the mode-matching technique effectively model both effects within a unified mathematical framework? To address these gaps, this study presents a comprehensive investigation of wave scattering in a bifurcated circular cylindrical waveguide, incorporating both reacting liners and step-discontinuities. The problem is formulated mathematically by defining impedance boundary conditions at the air-liner interface and solving the governing equations using the mode-matching (MM) technique. Unlike conventional approaches that rely on either analytical approximations or fully numerical solutions, our approach leverages eigenfunction expansions to achieve high accuracy while maintaining computational efficiency.The approach is followed by many authors to discuss physical situations, for instance see [27–30].

The novelty of this work lies in its integrated approach to modeling both liner effects and step-discontinuities within a single waveguide system. Thus, the study aims in two-fold: first to present the scattering analysis of step-discontinuity in coaxial waveguide; second to model and investigate the inclusion of acoustic liners in a bifurcated circular cylindrical waveguide. The applications of step-discontinuity with material contrast to model a silencing device is recently considered in [31–33]. Further, Peaks and Abrahams [34] analyzed scattering from bifurcated waveguide containing impedance conditions along the bounding walls. They assumed planar geometry and presented analysis based on WH technique. More recently Afzal *et al* [35] discussed the effects of step-discontinuity in bifurcated waveguide by using MM technique while Nawaz *et al* [36, 37] examined dynamical surfaces tailored in flexural shells considering the circular cylindrical waveguide. In present article step-discontinuity and/or liner conditions are assumed in circular cylindrical waveguide and solution is sorted by using MM technique. Key contributions include:

- Development of a unified mathematical model that incorporates reacting liners and step-discontinuities within a bifurcated waveguide framework.
- Application of the mode-matching technique to solve the acoustic boundary value problem with high accuracy and numerical stability.
- Validation of the solution through power conservation principles and matching condition reconstructions.
- Comprehensive parametric analysis demonstrating how liner impedance, discontinuity dimensions, and frequency variations influence wave scattering behavior.

By addressing these aspects, this study provides practical insights for designing advanced noise control devices and optimizing waveguide-based acoustic systems for enhanced performance.

In parallel with these developments, recent research in biomedical and magnetohydrodynamic (MHD) fluid mechanics has demonstrated the profound influence of wave dynamics, magnetic fields, and thermal effects on flow stability and transport properties. For instance, the interaction of magnetic fields and thermal radiation in ternary hybrid nanofluids has been studied to understand their effects in complex biomedical flows [38]. Similarly, studies on peristaltic slip flow in annular channels under variable magnetic fields have revealed how wave interactions, electro-osmotic effects, and nanoparticle aggregation influence transport phenomena [39, 40]. Additionally, the aggregation of nanoparticles and electro-osmotic propulsion in peristaltic nanofluid transport has provided insights into the role of wave-driven mechanisms in structured environments [41].

Following is the breakdown of the article: The problem is formulated mathematically in Sect 2, and the solution process is described in Sect 3. The the energy flux identities are discussed in Sect 4 while the results of the numerical experiments are presented in Sect 5. An overview and final observations are provided in Sect 6 as well.

## Mathematical formulation of the problem

**Fig 1:**
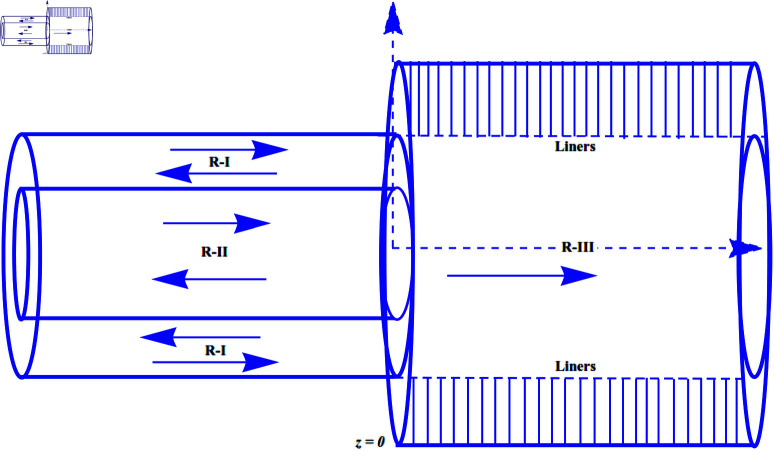
Bifurcated lined waveguide.

We consider acoustic scattering in a bifurcated cylindrical, infinite waveguide containing lining over annular region of locally reacting material. The inside of the waveguide is filled with compressible fluid, like air, having density ρ and sound speed *c*. The geometrical configuration as shown in Fig 1, is such that it comprises two coaxial circular regions at $\bar{z}\leq \bar{0}$ (regions I and II) connected to the lined region at $\bar{z}\geq \bar{0}$ (region III). The acoustic impedance of locally reacting material can be defined as [12]

$$\bar{Z}:=\frac{\bar{p}}{\bar{v}\cdot \bar{n}},$$ (1)

where $\bar{p}$ denotes acoustic pressure, $\bar{v}$ a velocity vector and $\bar{n}$ a unit normal vector directed into the lining. Note that the bared quantities denote dimensional setting of variables.

The incident excitations are introduced in Regions I and/or II, which function as the inlets of the guiding channel. This is the region where a fluid duct modes/mode will attenuate upon interaction with structural variations and linings of the region. To analyze the acoustic attenuation, a boundary value problem (BVP) in terms of fluid potential $\bar{\phi }$ is designed, where $\bar{p}=-\rho \partial \bar{\phi }/\partial \bar{t}$ and $\bar{v}=\bar{\nabla }\bar{\phi }$. Throughout the BVP, a time-harmonic $e^{-i\omega \bar{t}}$ dependence (in which ω is radian frequency) and the dimensionless transformations $r=k\bar{r}$, $t=\omega \bar{t}$ and $\phi =k^{2}\bar{\phi }/\omega$ are considered, where *k* is wave number. Consequently, in the duct regions, the time independent non-dimensional fluid potential ϕ(r,t) satisfies the Helmholtz equation

$$\left\{\frac{\partial^{2}}{\partial r^{2}}+\frac{1}{r}\frac{\partial }{\partial r}+\frac{\partial^{2}}{\partial z^{2}}+1\right\}\phi =0,$$ (2)

where $\phi =\phi_{j}$ for j=1,2 and 3 in regions I (R-I), II (R-II) and III (R-III), respectively. The bounding surfaces of regions I, II and III are acoustically rigid $(\bar{Z}=\infty$) and give ∂ϕ/∂r=0 at r=a,b, *z*<0 and r=d, *z*>0. However, for acoustic liner at r=b, *z*>0 the impedance $Z=\rho c\bar{Z}$ contains some finite value. The impedance of the liner can be found by considering a locally reactive honeycomb layer backed by rigid circular cavity. At *r* = *b*, the impedance of the liner backed by rigid cavity is

$$Z_{b}=i\frac{N_{0}^{\prime }(d)J_{0}(b)-N_{0}(b)J_{0}^{\prime }(d)}{N_{0}^{\prime }(d)J_{0}^{\prime }(b)-N^{\prime }k_{0}(b)J_{0}^{\prime }(d)},$$ (3)

where *J*_0_(.) and *N*_0_(.) are Bessel functions of first and second kind, respectively, and prime with these quantities denotes differentiation with respect to *r*. The liner conditions can be expressed mathematically as,

$$\phi_{3}-iZ_{b}\frac{\partial \phi_{3}}{\partial r}=0\;\text{at}\;r=b,\;z>0.$$ (4)

To analyze the scattering characteristics of liner conditions in bifurcated cylindrical configuration, the model problem is solved with and without liner conditions in subsequent sections.

## Solution methodology

Here the underlying problem is work out by using the MM technique which is very useful for calculating scattered fields when dealing with complex geometries and problems with high-order boundary conditions in ducts or channels, see for instance [29–31–42–44]. In order to determine behaviour of finite-length dissipative silencers, such as car mufflers, other numerical approaches like finite element or boundary element methods become computationally intensive as the excitation frequency and silencer dimensions rise. Although numerical approaches, such as the finite element method used by Gabard and Astley [44], provide flexibility for studying silencers of different sizes and forms, they provide computational hurdles when the problem’s degrees of freedom significantly rise, especially for large mufflers. An analytical or semi-analytical approach is preferable in these circumstances. Automobile mufflers have shown the MM technique to be effective, although in the case of zero mean flow. Finding a significant number of roots in the dispersion relationship is necessary to achieve analytical convergence in this approach, especially with regard to continuity conditions at the structure’s inlet and outflow planes. As a result, the MM solution is thought to be suitable and desirable for solving such problems.

The solution is sorted by determining the eigenfunction expansions of duct regions with the help of separation of variable technique, which yields

$$\phi_{1}(r,z)=\sum_{n=0}^{\infty }A_{n}R_{1}(\tau_{n},r)e^{i\eta_{n}z}+\sum_{n=0}^{\infty }B_{n}R_{1}(\tau_{n},r)e^{-i\eta_{n}z},$$ (5)

$$\phi_{2}(r,z)=\sum_{n=0}^{\infty }C_{n}R_{2}(\gamma_{n},r)e^{is_{n}z}+\sum_{n=0}^{\infty }D_{n}R_{2}(\gamma_{n},r)e^{-is_{n}z},$$ (6)

$$\phi_{3}(r,z)=\sum_{n=0}^{\infty }E_{n}R_{3}(\xi_{n},r)e^{i\lambda_{n}z},$$ (7)

where the quantities $R_{1}(\tau_{n},r)=J_{0}(\tau_{n},r)$, $R_{2}(\gamma_{n},r)=J_{0}(\gamma_{n}r)N_{0}^{߰}(\gamma_{n}a)-J_{0}^{߰}(\gamma_{n}a)N_{0}(\gamma_{n}r)$ and $R_{3}(\xi_{n},r)=J_{0}(\xi_{n},r)$ are the eigenfunctions of $n^{\text{th}}$ mode propagating in regions I, II, and III, respectively. The eigenvalues $\tau_{n}$, $\gamma_{n}$ and $\xi_{n}$ are the roots of characteristic equations

$$\kappa_{1}(\tau ,a):=J_{0}^{\prime }(\tau ,a)=0,$$ (8)

$$\kappa_{2}(\gamma ,a,b):=J_{0}^{\prime }(\gamma ,b)N_{0}^{\prime }(\gamma ,a)-J_{0}^{\prime }(\gamma ,a)N_{0}^{\prime }(\gamma ,b)=0$$ (9)

and

$$\kappa_{3}(\xi ,b,d):=\left\{ \begin{array}{c} J_{0}(\xi ,b)-iZ_{b}J_{0}^{\prime }(\xi ,b)=0,\;\text{with liner conditions}\\ J_{0}^{\prime }(\xi ,d)=0,\;\;\;\;\text{without liner conditions}. \end{array}\right.$$ (10)

There are infinite many values of (τ,γ,ξ) for which the aforementioned relations hold. These values can be determined numerically, and reveal the mode wavenumbers $\eta_{n}=\sqrt{1-\tau_{n}^{2}},$ $s_{n}=\sqrt{1-\gamma_{n}^{2}}$ and $\lambda_{n}=\sqrt{1-\xi_{n}^{2}}.$ The waveguide contains incident radiations from region I and/or II, and the first terms on right hand sides of Eq (5) and Eq (6) specify these radiations. The eigenfunctions in planar geometries are linearly independent and satisfy generalized orthogonality, which facilitates the transformation of differential systems into linear algebraic systems. This conversion ensures that the unknown dependent sums are selected to guarantee the convergence of the generalized Fourier series. A detailed analysis of this convergence can be found in [45]. Note that the eigenfunctions $R_{1}(\tau_{n},r)$, $R_{2}(\gamma_{n},r)$ and $R_{3}(\xi_{n},r)$ are orthogonal in nature and satisfy the orthogonality relations

$$\int_{0}^{a}R_{1}(\tau_{m},r)R_{1}(\tau_{n},r)rdr=\delta_{mn}F(\tau_{n},a),\;\int_{a}^{b}R_{2}(\gamma_{m},r)R_{2}(\gamma_{n},r)rdr=\delta_{mn}G_{n},$$ (11)

$$\int_{0}^{b}R_{3}(\xi_{m},r)R_{3}(\xi_{n},r)rdr=\delta_{mn}F(\xi_{n},b),\;\int_{0}^{d}R_{3}(\xi_{m},r)R_{3}(\xi_{n},r)rdr=\delta_{mn}F(\xi_{n},d),$$ (12)

where


$$F(x,r):=\frac{r^{2}}{2}J_{0}^{2}(xr)$$

and


$$G_{n}\;=\frac{1}{2}[b^{2}\backslash \{(J_{0}^{2}(b\gamma_{n})+J_{1}^{2}(b\gamma_{n}))-2J_{1}(a\gamma_{n})N_{1}(a\gamma_{n})(J_{0}(b\gamma_{n})N_{0}(b\gamma_{n})\mathrm{\backslash notag}\;\;+J_{1}(b\gamma_{n})N_{1}(b\gamma_{n}))+J_{1}^{2}(a\gamma_{n})(N_{0}^{2}(b\gamma_{n})+N_{1}^{2}(b\gamma_{n}))\backslash \}-\frac{4}{\pi^{2}\gamma_{n}}].$$

For the fluid mode incident with unit amplitudes from regions I and II, the values of coefficients become $A_{n}=\delta_{n0}$ and $C_{n}=\delta_{n0}$, respectively. Whereas, the scattering coefficients $\{B_{n},D_{n},E_{n}\},n=0,1,2,\cdots$ remain unknowns. To find these unknowns we apply matching conditions over pressures and velocities at interface. At z=0, the continuity of pressures across the regions I, II and III can be stated as

$$\phi_{1}(r,0)=\phi_{3}(r,0),\;0\leq r\leq a$$ (13)

and

$$\phi_{2}(r,0)=\phi_{3}(r,0),\;a\leq r\leq b.$$ (14)

On multiplying Eq (13) with $rR_{1}(\tau_{m},r)$, integrating over 0<*r*<*a* and Eq (14) with $rR_{2}(\gamma_{m},r)$, integrating over *a*<*r*<*b*, we get

$$\int_{0}^{a}\phi_{1}(r,0)R_{1}(\tau_{m},r)rdr=\int_{0}^{a}\phi_{3}(r,0)R_{1}(\tau_{m},r)rdr$$ (15)

and

$$\int_{a}^{b}\phi_{2}(r,0)R_{2}(\gamma_{m},r)rdr=\int_{a}^{b}\phi_{3}(r,0)R_{2}(\gamma_{m},r)rdr.$$ (16)

By substituting Eq (5)–Eq (7) into Eq (15)–Eq (16) and simplifying with the aid of Eq (11) and Eq (12), it is, respectively, found that

$$B_{m}=-A_{m}+\frac{1}{F(\tau_{m},a)}\sum_{n=0}^{\infty }E_{n}Q_{mn},$$ (17)

and

$$D_{m}=-C_{m}+\frac{1}{G_{m}}\sum_{n=0}^{\infty }E_{n}P_{mn},$$ (18)

where

$$Q_{mn}=\int_{o}^{a}R_{1}(\tau_{m},r)R_{3}(\xi_{m},r)rdr,\;P_{mn}=\int_{a}^{b}R_{2}(\gamma_{m},r)R_{3}(\xi_{m},r)rdr.$$ (19)

Now to find *E*_*n*_, we apply the continuity of velocity conditions at interface *z* = 0. As the region III contains a cavity that may or may not comprise a filling of acoustic liners. The normal velocity condition on both the cases is discussed in subsequent subsections.

### Liners condition

When that the geometrically discontinuous portion of region III is acoustic liners conditions, the normal velocity of this is related to regions I and II at *z* = 0 as

$$\phi_{3z}(r,0)=\left\{ \begin{array}{c} \phi_{1z}(r,0)\;\;0\leq r\leq a\\ \phi_{2z}(r,0)\;\;a\leq r\leq b. \end{array}\right.$$ (20)

On multiplying Eq (20) with $rR_{3}(\xi_{m},r)$, integrating over 0<*r*<*b*, it is found that

$$\int_{0}^{b}\phi_{3z}(r,0)R_{3}(\xi_{m},r)rdr=\int_{0}^{a}\phi_{1z}(r,0)R_{3}(\xi_{m},r)rdr+\int_{a}^{b}\phi_{2z}(r,0)R_{3}(\xi_{m},r)rdr$$ (21)

By invoking Eq (5)-Eq (7) into Eq (21) and simplifying with the aid of Eq (12), it is straightforward to write

$$E_{m}=\frac{1}{F(\xi_{m},b)\lambda_{m}}\left(\sum_{n=0}^{\infty }\{A_{n}-B_{n}\}\eta_{n}Q_{nm}+\sum_{n=0}^{\infty }\{C_{n}-D_{n}\}s_{n}P_{nm}\right),$$ (22)

In this way, we get a linear algebraic system defined by Eq (16), Eq (17) and Eq (22). The system is truncated and inverted for required unknowns.

### Step-discontinuity

Here we discuss the case when the geometrically discontinuous portion of region III does not involve any liner conditions. Then the normal velocity of region III is related to regions I and II at *z* = 0 as

$$\phi_{3z}(r,0)=\left\{ \begin{array}{c} \phi_{1z}(r,0)\;\;0\leq r\leq a\\ \phi_{2z}(r,0)\;\;a\leq r\leq b\\ 0\;\;\;\;b\leq r\leq d \end{array}\right.$$ (23)

On multiplying Eq (23) with $rR_{3}(\xi_{m},r)$, integrating over 0<*r*<*d*, it is found that

$$\int_{0}^{d}\phi_{3z}(r,0)R_{3}(\xi_{m},r)rdr=\int_{0}^{a}\phi_{1z}(r,0)R_{3}(\xi_{m},r)rdr+\int_{a}^{b}\phi_{2z}(r,0)R_{3}(\xi_{m},r)rdr$$ (24)

By substituting Eq (5)-Eq (7) into Eq (24) and simplifying with the aid of Eq (12), we get

$$E_{m}=\frac{1}{F(\xi_{m},d)\lambda_{m}}\left(\sum_{n=0}^{\infty }\{A_{n}-B_{n}\}\eta_{n}Q_{nm}+\sum_{n=0}^{\infty }\{C_{n}-D_{n}\}s_{n}P_{nm}\right).$$ (25)

For air filled region, we achieve the linear algebraic system defined by Eq (16), Eq (17) and Eq (25). The system is truncated and inverted for required unknowns.

## Energy flux identities

The energy flux propagating in the duct regions is discussed in this section. The non-dimensional form of energy flux is [11]

$$\text{Energy flux}=\text{Re}\left[\int_{\Omega }i\phi {\left(\frac{\partial \phi }{\partial z}\right)}^{*}rdr\right],$$ (26)

where (*) denotes the complex conjugate and Ω expresses the bounding domain.

For the fluid mode incident with unit amplitudes from regions I and II, the values of energy flux are found to be $\varepsilon_{\text{I}}^{i}=a^{2}/2$ and $\varepsilon_{\text{II}}^{i}=(b^{2}\;-\;a^{2})/2$, respectively. Whereas, the reflected flux in regions I and II are respectively found to be

$$\varepsilon_{\text{I}}^{r}=-\text{Re}\left[\sum_{n=0}^{\infty }|B_{n}{|}^{2}\eta_{n}F(\tau_{n},a)\right]\;\text{and}\;\varepsilon_{\text{II}}^{r}=-\text{Re}\left[\sum_{n=0}^{\infty }|D_{n}{|}^{2}s_{n}G_{n}\right].$$ (27)

Note that the negative sign in Eq (27) represents the propagation of energy flux towards negative *z* direction. If there is no dissipation of energy in region III such as the case when the region III is filled with air, the transmitted energy flux is calculated as

$$\varepsilon_{\text{III}}^{t}=\text{Re}\left[\sum_{n=0}^{\infty }|E_{n}{|}^{2}\lambda_{n}F(\xi_{n},d)\right].$$ (28)

For the air filled cavity, the energy flux identity can be established on the principle of conservation of energy flux, that gives

$$\varepsilon_{\text{I}}^{i}+\varepsilon_{\text{II}}^{i}=\varepsilon_{\text{I}}^{r}+\varepsilon_{\text{II}}^{r}+\varepsilon_{\text{III}}^{t}.$$ (29)

For convenience in physical analysis, one can scale the incident energy flux over unity by dividing both sides of Eq (29) with $\varepsilon_{\text{I}}^{i}+\varepsilon_{\text{II}}^{i}$ to get

$$1=\varepsilon_{1}+\varepsilon_{2}+\varepsilon_{3},$$ (30)

where $\varepsilon_{1}=\varepsilon_{\text{I}}^{r}/(\varepsilon_{\text{I}}^{i}+\varepsilon_{\text{II}}^{i})$, $\varepsilon_{2}=\varepsilon_{\text{II}}^{r}/(\varepsilon_{\text{I}}^{i}+\varepsilon_{\text{II}}^{i})$ and $\varepsilon_{3}=\varepsilon_{\text{III}}^{t}/(\varepsilon_{\text{I}}^{i}+\varepsilon_{\text{II}}^{i}).$ The energy identity (30) can also be formulated by using the expressions of scattering unknowns. For instance, on multiplying (25) with $\sum_{m=0}^{\infty }E_{m}^{*}$, we achieve

$$\sum_{m=0}^{\infty }|E_{n}{|}^{2}F(\xi_{m},d)\lambda_{m}=\sum_{n=0}^{\infty }\{A_{n}-B_{n}\}\eta_{n}\sum_{m=0}^{\infty }E_{m}^{*}Q_{nm}+\sum_{n=0}^{\infty }\{C_{n}-D_{n}\}s_{n}\sum_{m=0}^{\infty }E_{m}^{*}P_{nm}.$$ (31)

For air filled case, the eigenvalues $\{\tau_{n},\gamma_{n},\xi_{n}\},n=0,1,2.\cdots$, are real, therefore, from Eq (17) and Eq (18) we find

$$\sum_{m=0}^{\infty }E_{m}^{*}Q_{nm}=(A_{n}^{*}+B_{n}^{*})F(\tau_{n},a),$$ (32)

and

$$\sum_{m=0}^{\infty }E_{m}^{*}P_{nm}=(C_{n}^{*}+D_{n}^{*})G_{n}$$ (33)

On using Eq (32) and Eq (33) into Eq (33) and then collecting the real parts, it is found that


$$\sum_{m=0}^{\infty }|E_{n}{|}^{2}F(\xi_{m},d)\lambda_{m}+\sum_{m=0}^{\infty }|B_{n}{|}^{2}F(\xi_{m},a)\eta_{m}+\sum_{m=0}^{\infty }|D_{n}{|}^{2}G_{m}s_{m}$$

$$=\sum_{n=0}^{\infty }\{|A_{n}{|}^{2}+\;A_{n}B_{n}^{*}-B_{n}A_{n}^{*}\}F(\tau_{n},a)\eta_{n}+\sum_{n=0}^{\infty }\{|C_{n}{|}^{2}+\;C_{n}D_{n}^{*}-D_{n}C_{n}^{*}\}G_{n}s_{n}.$$ (34)

For fluid modes from regions I and II, we consider $A_{n}=\delta_{n0}$ and $C_{n}=\delta_{n0}$, which on substituting into Eq (34), and then collecting the real parts of resulting, we find Eq (29).

## Numerical results and discussions

In this section, the numerical experiments are performed and discussed. First, the truncation of linear algebraic systems by fixing the associated parameters m=n=0,1,2⋯N terms is made, and then the retained systems are solved numerically. For air filled problem, the linear algebraic system is given by Eq (16), Eq (17) and Eq (25), whilst, for acoustic liners problem the linear algebraic system is defined by Eq (16), Eq (17) and Eq (22).

The validity of mode-matching solution is an inherit aspect the technique. The solution is projected on the orthogonal basis functions having distinct eigenvalues. With the aid of appropriate orthogonality relations, the differential systems are converted into linear algebraic systems of infinite equations. The systems are truncated, and then are solved numerically. Nevertheless, the associated eigenfunctions are complete and linearly independent which ensure the pointwise convergence of eigenfunction expansions Eq (5)–Eq (7). However, to confirm the adequate convergence of truncated form of solutions, the matching conditions Eq (13)–Eq (14), Eq (20) and Eq (23) are numerically reconstructed by using the truncated solution. Further, the energy flux identity Eq (30) is formulated, and then is analyzed with truncated form of solutions whose satisfaction confirms the accuracy of performed algebra as well as the truncated solution.

**Fig 2:**
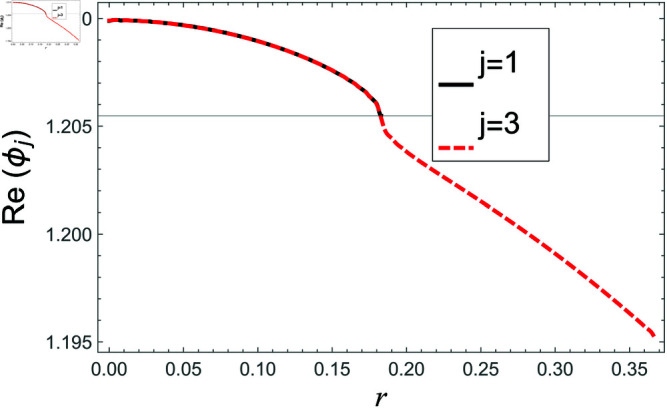
Acoustic liners containing problem: Real parts of pressure vs. duct radius at *z* = 0 with frequency 100Hz and truncation parameter *N* = 80 terms.

**Fig 3:**
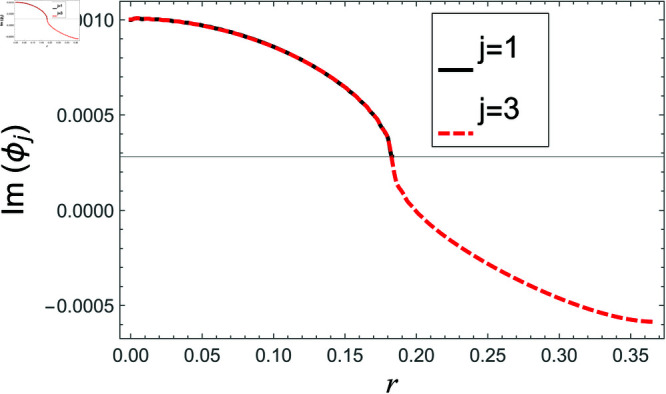
Acoustic liners containing problem: Imaginary parts of pressure vs. duct radius at *z* = 0 with frequency 100Hz and truncation parameter *N* = 80 terms.

**Fig 4:**
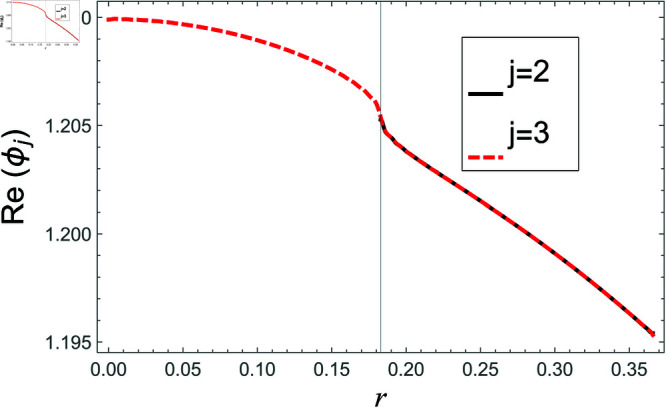
Acoustic liners containing problem: Real parts of pressure vs. duct radius at *z* = 0 with frequency 100Hz and truncation parameter *N* = 80 terms.

**Fig 5:**
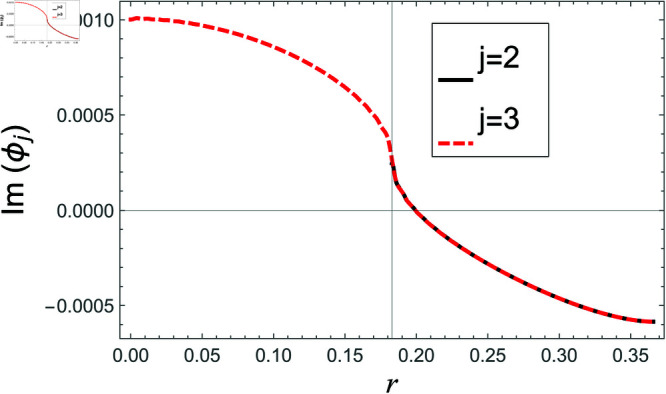
Acoustic liners containing problem: Imaginary parts of pressure vs. duct radius at *z* = 0 with frequency 100Hz and truncation parameter *N* = 80 terms.

**Fig 6:**
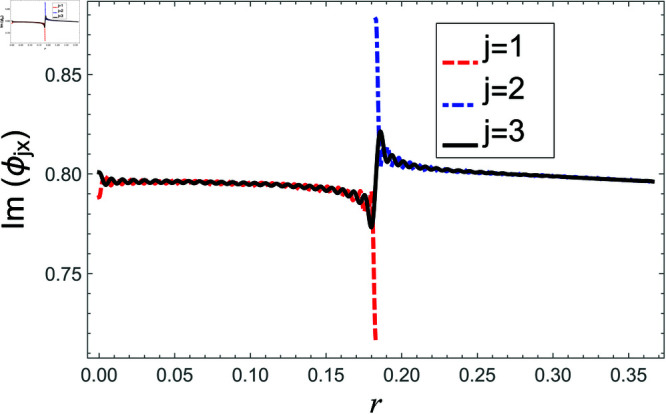
Acoustic liners containing problem: Real parts of normal velocity vs. duct radius at *z* = 0 with frequency 80Hz and truncation parameter *N* = 80 terms.

**Fig 7:**
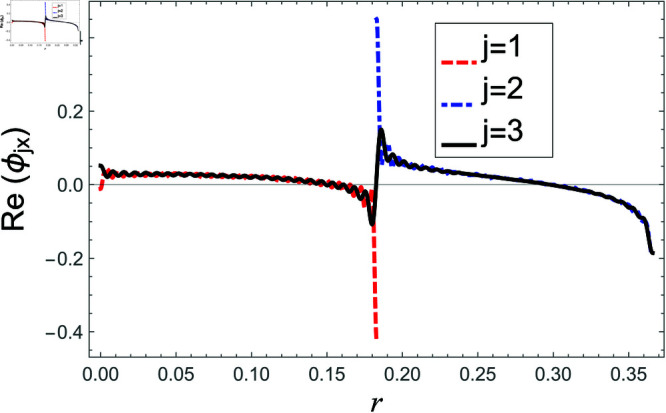
Acoustic liners containing problem: Imaginary parts of normal velocity vs. duct radius at *z* = 0 with frequency 80Hz and truncation parameter *N* = 80 terms.

**Fig 8:**
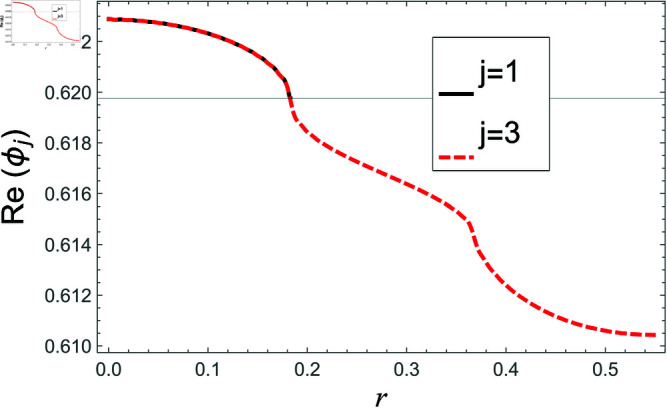
Air containing problem: Real parts of pressure vs. duct radius at *z* = 0 with frequency 100Hz and truncation parameter *N* = 80 terms.

**Fig 9:**
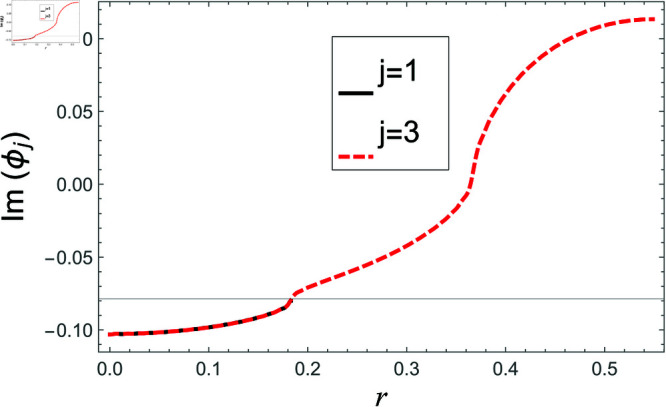
Air containing problem: Imaginary parts of pressure vs. duct radius at *z* = 0 with frequency 100Hz and truncation parameter *N* = 80 terms.

**Fig 10:**
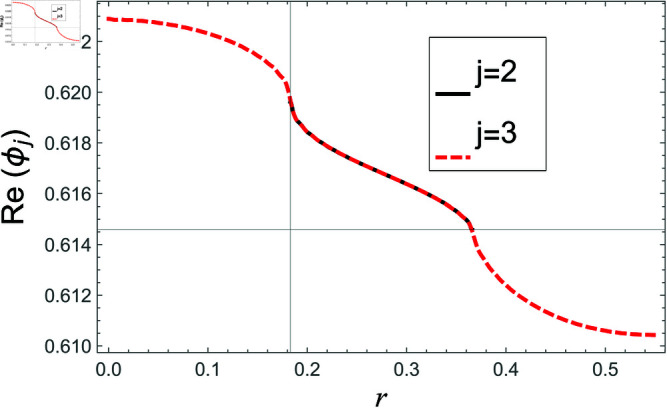
Air containing problem: Real parts of pressure vs. duct radius at *z* = 0 with frequency 100Hz and truncation parameter *N* = 80 terms.

**Fig 11:**
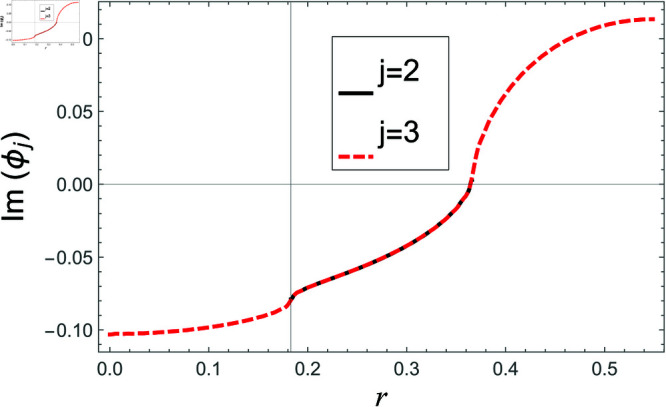
Air containing problem: Imaginary parts of pressure vs. duct radius at *z* = 0 with frequency 100Hz and truncation parameter *N* = 80 terms.

**Fig 12:**
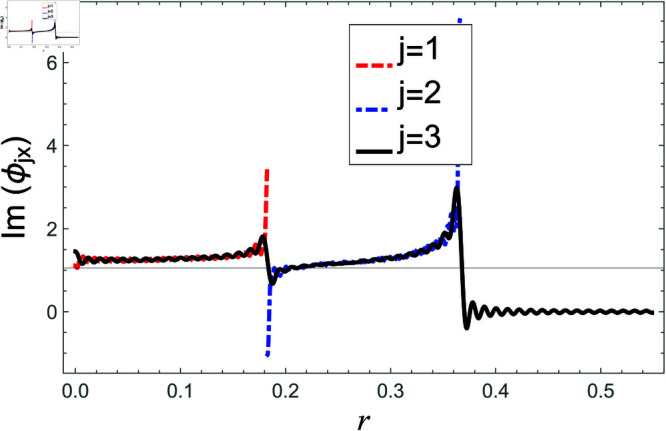
Air containing problem: Real parts of normal velocity vs. duct radius at *z* = 0 with frequency 100Hz and truncation parameter *N* = 80 terms.

**Fig 13:**
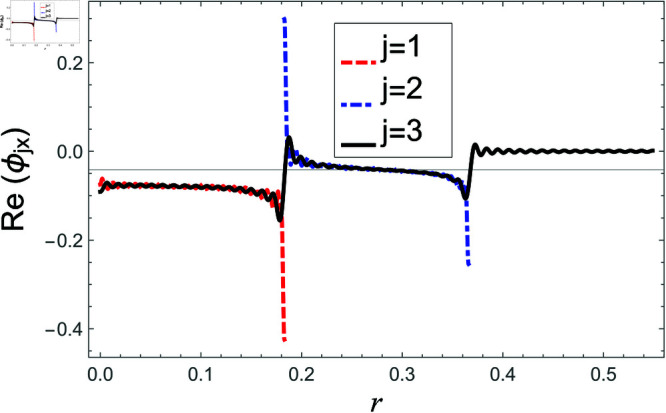
Air containing problem: Imaginary parts of normal velocities vs. duct radius at *z* = 0 with frequency 100Hz and truncation parameter *N* = 80 terms.

Figs 2–13 show the graphs of matching conditions achieved with the truncated form of solutions. The numerical computations are performed by fixing radii as; $\bar{a}=0.1$m, $\bar{b}=0.2$m, and $\bar{d}=0.3$m, where the sound speed, frequency, density of the air, number of terms are assumed to be *c* = 344m/s, *f* = 100Hz, ρ=1.2 kg/${\text{m}}^{3}$ and *N* = 80 terms, respectively. For acoustic liners containing problem, the continuity of pressures at normal interface *z* = 0 is shown in Figs 2–5, whereas, the continuity of normal velocities is displayed in Figs 6–7.

The real and imaginary parts of pressures in regions I and II match exactly to respective real and imaginary part of pressure in region III at interface *z* = 0., for instance see Figs 2–5. It reflects numerically the conditions Eq (13) and Eq (14). Accordingly, the matching of normal velocity of region III to the normal velocities of regions I and II is displaced in Figs 6–7. It is seen that the real and imaginary parts of normal velocity of region III match exactly to the respective velocities of regions I and II, which numerically reflect the satisfaction of condition Eq (20).

For air containing problem, the continuity of pressures and normal velocities at interface *z* = 0 are shown in Figs 8–13, respectively. From Figs 8–11, it is found that the real and imaginary parts of pressures in regions I and II match exactly to respective real and imaginary part of pressure in region III at interface *z* = 0., which exactly reflects numerically the continuity conditions of pressures Eq (13) and Eq (14).

Likewise, the continuity condition of normal velocities Eq (23) is numerically depicted in Figs 12–13. Clearly, the real and imaginary components of normal velocity of region III match exactly to the respective velocities of regions I and II in their associated regimes but becomes zero when *b*<*r*<*d*, which, in fact, concludes the reconstruction of the normal velocity condition Eq (23) numerically.

**Fig 14:**
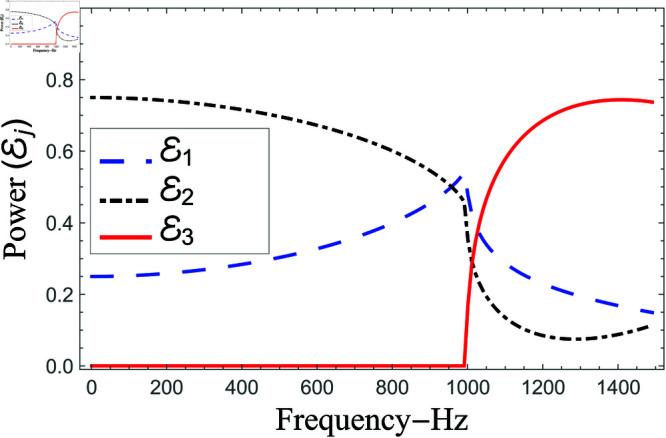
Scattering powers against frequency for acoustic liners filled cavity where $\bar{a}=0.05$m, $\bar{b}=0.1$m and $\bar{d}=0.15$m.

**Fig 15:**
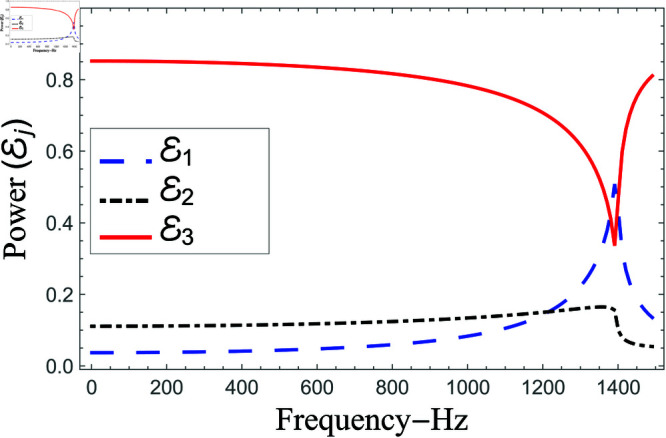
Scattering powers against frequency for air filled cavity, where $\bar{a}=0.05$m, $\bar{b}=0.1$m and $\bar{d}=0.15$m.

**Fig 16:**
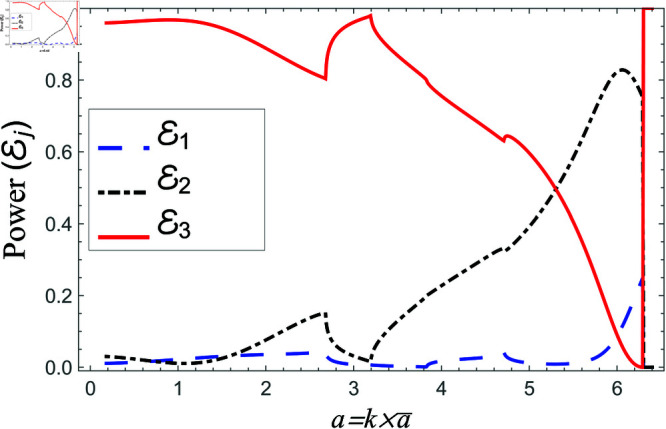
Scattering powers against non-dimensional radius a for acoustic liners filled cavity, where $0.01m\leq \bar{a}\leq 0.35$m, $\bar{b}=2\bar{a}$ and $\bar{d}=2.5\bar{a}$.

**Fig 17:**
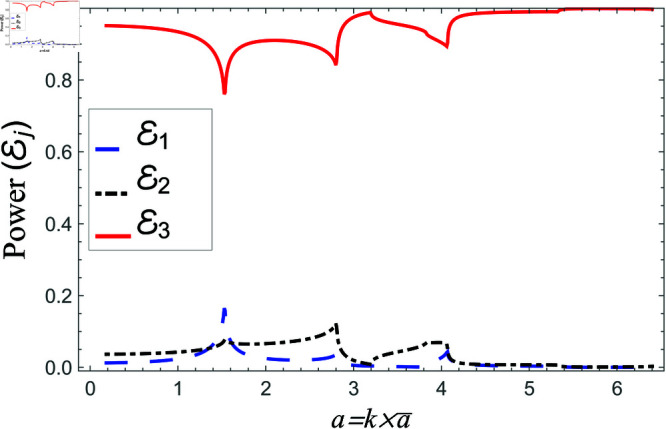
Scattering powers against non-dimensional radius a for air filled cavity, where $0.01m\leq \bar{a}\leq 0.35$m, $\bar{b}=2\bar{a}$ and $\bar{d}=2.5\bar{a}$.

**Fig 18:**
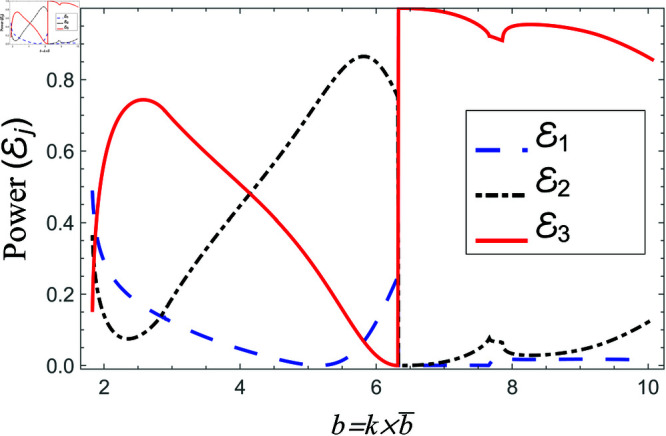
Scattering powers against non-dimensional radius b for acoustic liners filled cavity, where $\bar{a}=0.5\bar{b}$, $0.1<\bar{b}<0.45$m and $\bar{d}=1.5\bar{b}$.

**Fig 19:**
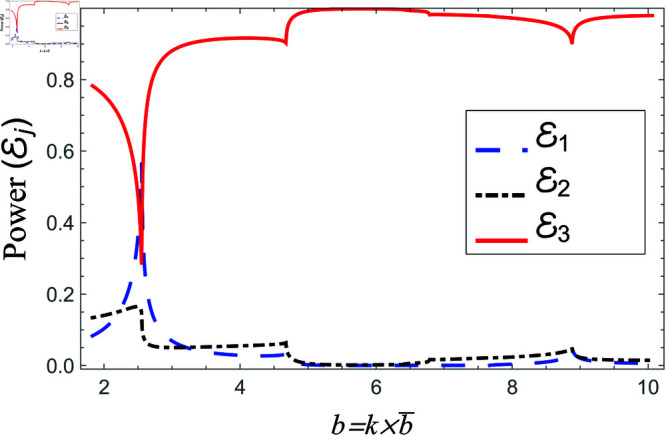
Scattering powers against non-dimensional radius b for air filled cavity, where $\bar{a}=0.5\bar{b}$, $0.1<\bar{b}<0.45$m and $\bar{d}=1.5\bar{b}$.

**Fig 20:**
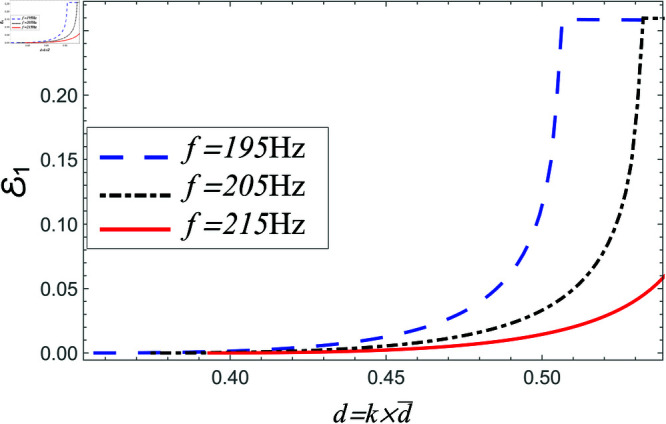
Power reflected in region I vs. step-discontinuity for acoustic liners filled cavity, where $\bar{a}=0.05$m, $\bar{b}=0.1$m and $\bar{b}<\bar{d}<0.15$m.

**Fig 21:**
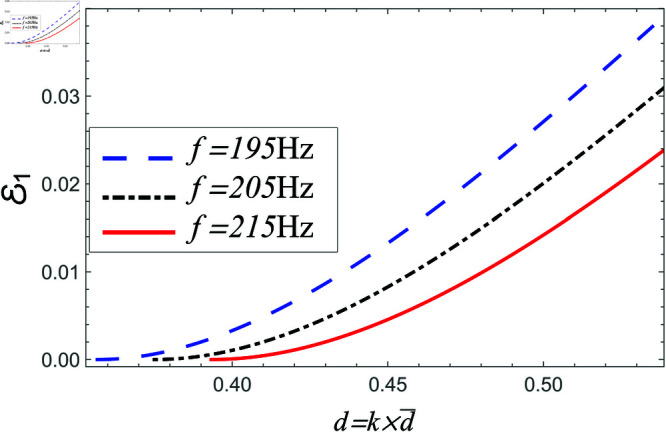
Power reflected in region I vs. step-discontinuity for air filled cavity, where $\bar{a}=0.05$m, $\bar{b}=0.1$m and $\bar{b}<\bar{d}<0.15$m.

**Fig 22:**
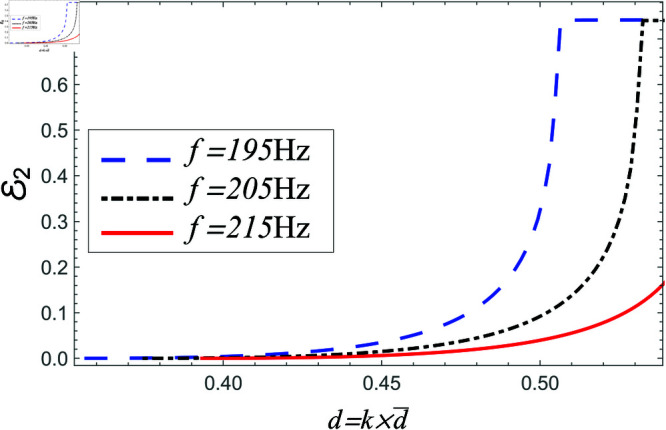
Power reflected in region II vs. step-discontinuity for acoustic liners filled cavity, where $\bar{a}=0.05$m, $\bar{b}=0.1$m and $\bar{b}<\bar{d}<0.15$m.

**Fig 23:**
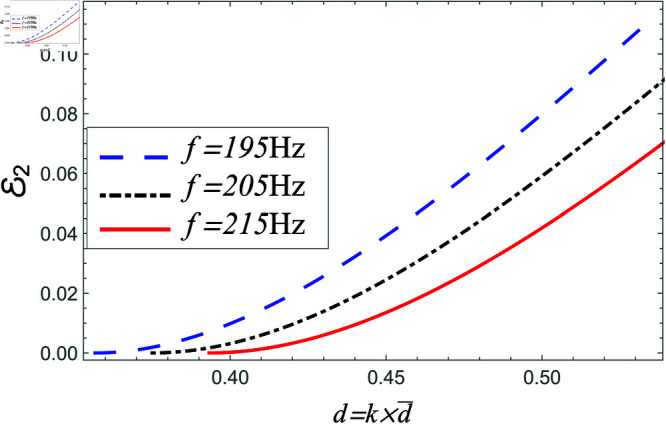
Power reflected in region II vs. step-discontinuity for air filled cavity, where $\bar{a}=0.05$m, $\bar{b}=0.1$m and $\bar{b}<\bar{d}<0.15$m.

**Fig 24:**
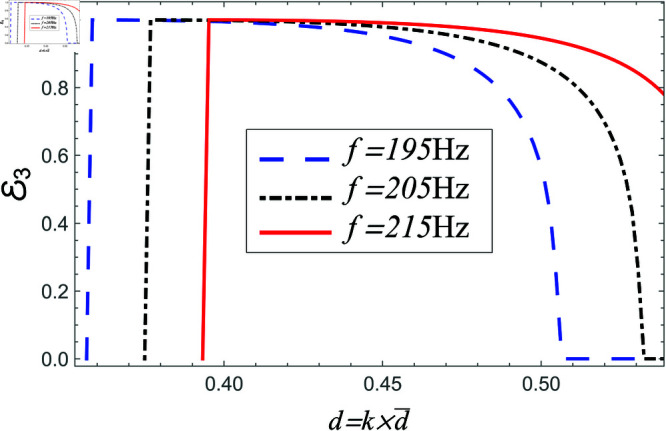
Power transmitted in region III vs. step-discontinuity for acoustic liners filled cavity, where $\bar{a}=0.05$m, $\bar{b}=0.1$m and $\bar{b}<\bar{d}<0.15$m.

**Fig 25:**
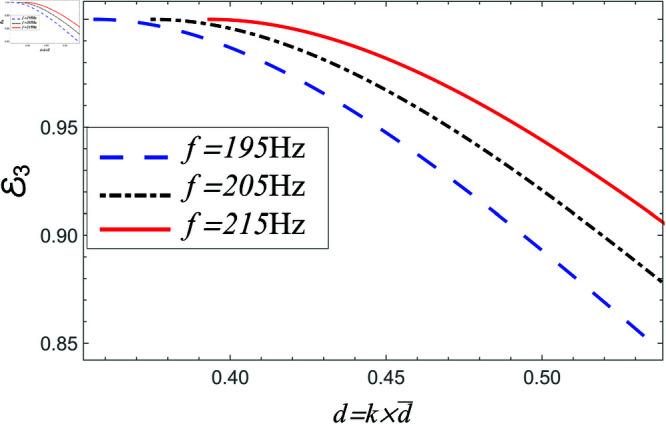
Power transmitted in region III vs. step-discontinuity for air filled cavity, where $\bar{a}=0.05$m, $\bar{b}=0.1$m and $\bar{b}<\bar{d}<0.15$m.

Consequently, the truncated form of solutions satisfy the matching conditions. Nevertheless, there occur some oscillations in the graphs of normal velocities and the amplitude of oscillations increases significantly as r→b. This is because of the singular behavior of the velocity field at the corner (r,z)=(b,0), reinforcing the need for mode-matching validation. This is confirmed by ensuring sufficient terms in the truncation for modal coefficient convergence and accurate reconstruction of matching conditions. Nawaz and Lawrie [32] studied a similar system with stronger singularities, using 80 terms for fundamental mode forcing and 200 for higher modes. Given the weaker singularity in the current case, good convergence is achieved even with fewer terms. Furthermore, the truncated form of solutions satisfy the conserve power identity Eq (30), for instance see Figs 14–25. More so, the MM solution outlined above not only preserves power equilibrium but also adheres to eigen properties, as detailed by Lawrie [45], while effectively satisfying pressure and normal velocity flux, even in scenarios involving corner singularities. This certainly validates the solution to the governing problems.

To investigate the effects of liners insertion and exclusion on scattering Figs 14–25 are plotted. Figs 14–15 contain the reflected and transmitted powers against frequency while the dimensions of guiding structure remain fixed at $\bar{a}=0.05$m, $\bar{b}=0.1$m and $\bar{d}=0.15$m, and solutions are truncated with *N* = 80 terms. From the curves shown in Fig 14, it is observed that in low frequency regime whereby *f*<997Hz, the radiated power completely goes on reflection and there is no transmission. However, as the first cut-on frequency of region III 997 Hz appears, the scatting is swapped such that upto 80 percent of the energy flux enters into the region III, and rest into regions I and II. However, in the absence of acoustic liners (air filled cavity) such transmission loss is not evident, see for instance Fig 15. The fluid borne modes of all regions are cut-on at *f* = 1 Hz, and all of the duct regions propagate energy from start, nonetheless 80 percent of the energy enters into region III and rest into I and II. However, a dip like decrement appears as the second mode of duct III tends to cut-on limit at *f* = 1385Hz. These findings show the importance of liners in modifying the acoustic response of the waveguide. Liners influence modal interactions by changing impedance conditions, resulting in frequency-dependent transmission behavior. The findings also show how geometric discontinuities and impedance variations affect energy flux distribution, highlighting the need of correctly adjusting liner qualities for successful acoustic wave control.

Figs 16–17 depict the variation of scattering energies verses non-dimensional radius $a=k\bar{a}$ of region I when $0.01\text{m}\leq \bar{a}\leq 0.35$m, $\bar{b}=2\bar{a}$ and $\bar{d}=2.5\bar{a}$m. For liners problem, the transmitted energy flux decreases from maximum to zero at *a* = 6.3 with up and down dips, see for instance Fig 16. The dips appear due of occurrence of cut-on points of regions I, II and III, whereas, the zero transmission loss takes place when the cut-on points of regions II and III coincide. Note that the cut-on points of region I occur at a=0.18,3.83,, region II at a=0.18,3.29,6.3 and region III at a=0.18,2.68,4.72,6.3. On the other hand, in the absence of liners (air filled case) the transmitted power remains at maximum in whole regime along with spikes appearing as *b* approaches to cut-on point, see for instance Fig 17. The cut-on points in air-filled case for region I are at b=1.82,7.68, for region II are at b=1.82,6.4 and region III are at b=1.82,6.4,7.86. These findings highlight the effects of waveguide geometry and liner conditions on acoustic scattering behavior. Liners introduce frequency-dependent attenuation effects, which selectively suppress transmission under specific resonance situations. Meanwhile, in the air-filled case, the system is more transparent to wave propagation, with transmission determined solely by the waveguide’s intrinsic cut-on properties. This approach sheds light on how to optimize liner topologies for effective energy distribution in actual applications.

Figs 18-19 comprise the effects of non-dimensional radius $b=k\bar{b}$ on scattering, where $0.1\text{m}\leq \bar{b}\leq 0.45$m, $\bar{a}=0.5\bar{b}$ and $\bar{d}=1.5\bar{b}$m. For liners containing problem, the transmitted energy flux decreases from about eighty percent to zero at *b* = 6.4, and then suddenly it goes to its peak value. This point is basically the second cut-on point of regions II and III. After this point, the transmission slightly decreases with a dip at next cut-on of region II. Nevertheless, overall transmission remains at maximum value the reflected component of region II behaves conversely to the transmitted component of region III, see for instance Fig 18. Note that the cut-on points of region I occur at b=1.83,7.68, region II at b=1.83,6.4,7.86 and region III at b=1.83,6.4. On the other hand, for air filled case the transmitted power decreases from 80 percent to about 30 percent and then increases by increasing *b*, whereas, the reflecting powers behave conversely, for instance see Fig 19. The cut-on points in air-filled case for region I are at b=1.82,7.49, for region II are at b=1.82,2.56,4.75,6.95 and region III are at b=1.82,6.49. Further, the transmission curve remains along the peak with slight dips at cut-on points and reflected curves along the bottom such that the sum of all power components is unity at each value. These findings highlight the effect of liner presence on mode excitation and energy transfer, revealing that liners can efficiently control transmission characteristics by suppressing certain frequency ranges while promoting wave propagation in others. The observed dips and peaks in the transmission curves correlate to mode transitions, highlighting the importance of cut-on frequencies in determining overall scattering behavior.

Figs 20-25 depict the variation in scattering powers verses step-discontinuity at different values of frequency, where $\bar{a}=0.05$m, $\bar{a}=0.1$ and $\bar{b}<\bar{d}=1.5$m. In Figs 20–21, the power reflecting in region I against non-dimensional height varying from $\bar{d}=\bar{b}=0.1$m to $\bar{d}=1.5$m at frequency 195 Hz, 205 Hz and 215 Hz. It is seen that by increasing the height of vertical step the reflection in duct region I is increased. The variation in more evident at lower frequency and have lesser amount at higher frequency. However, for air filled case, the magnitude of reflected air energy flux is much less than the amount with liners including duct, for instance see Fig 20 and Fig 21.

In Figs 22–23, the power propagating in region II against step discontinuity with different values of frequency is shown. The observations are portrayed at the 195 Hz, 205 Hz and 215 Hz. From the comparison of Figs 20–23, it is found that the magnitude of reflecting power towards region II is more than the magnitude of power in region I. The reason behind is the participation of higher order cut-on modes in region II. The phenomenon is present in liners comprising problem as well as in the air containing problem.

In Figs 24–25, the power transmitting in region III against step discontinuity with different values of frequency is displayed. It is observed for planar structure majority of power is transmitted and reflection behaves conversely with liners as well as air filled settings. However, for non-planar structure there appear some reflection and transmission due to the involvement of vertical step discontinuity. For air filled case, as we increase the height of vertical step the transmission in region III decreases, and the reflection in regions I and II increases, see for instance Fig 25. However, in liners containing problem the transmission is enhanced more rapidly against step-discontinuity as compared with air filled cases. Moreover, by increasing frequency the transmission is increased and reflection is decreased. Overall, these findings emphasize the importance of both step geometry and liner conditions in determining wave energy distribution, especially when vertical discontinuities influence modal interactions and scattering behavior.

It is clear from the preceding numerical experiments that changes in material qualities and structural discontinuities have a considerable impact on the scattering powers. Further, the truncated solution satisfies matching conditions, achieving energy balance at N=80 terms. Increasing terms enhances convergence, though scattering power results remain consistent with fewer modes. While higher terms improve matching condition reconstruction, they also increase computational time.

## Summary

The reflection and transmission from a coaxial waveguide by reacting liners and/or geometric discontinuity is studied. In boundary value problem, the reacting liners appear as an impedance form of boundary condition and it includes the specific impedance defined on interface between air and liners. The physical problems having incident radiations from inner and annular circular cylindrical regions are solved by applying MM technique. The technique is well established and relies on the orthogonal characteristics of eigenfunctions of duct regions. These orthogonal properties help to convert the differential system into linear algebraic systems, which are then truncated and solved numerically. Further, a variety of numerical experiment are performed to analyze the effect of reacting liners and step-discontinuity on scattering. It is found that the reacting liners and step-discontinuity have a significant impact on scattering against frequency as well as duct dimensions. It has been concluded that:

- The reacting liners attenuate fluid borne mode in such a way that no transmission occurs against frequency upto the value where the secondary mode becomes cut-on. However, with step-discontinuity case, the observation is reversed.
- By changing the radii of circular regions the scattering can be optimized. The device is more operative with liner conditions as compared with step-discontinuity.
- By varying the size of liners or step-discontinuity the reflection and transmission of energy flux are changed. By increasing the values of frequency the scattering behavior is enhanced.
- Planar and non-planar structures behave differently in terms of power transmission and reflection when there is a step discontinuity. Planar structures primarily exhibit transmission, with liners and air-filled cases showing a contrastive reflection. The main cause of reflection and transmission in non-planar structures is vertical step discontinuity. In air-filled cases, raising the step height increases reflection and decreases transmission, but liners greatly increase transmission.
- The MM solution maintains power balance and adheres to eigen properties while effectively satisfying pressure and normal velocity flux, thereby explicitly validating the solution to the governing problems.

## References

[1] Mangiarotty RA. Acoustic lining concepts and materials for engine ducts. J Acoust Soc Am. 1970;48(3C):783–94.

[2] Martin V, Cummings A, Gronier C. Discrimination of coupled structural/acoustical duct modes by active control: principles and experimental results. J Sound Vib. 2004;274:583–603.

[3] Feng X, Wang X, Zhao S. Vibration control and robustness analysis of tensegrity structures via fuzzy dynamic sliding mode control method. Structures. 2024;67:106931.

[4] Nguyen-Thai V, Ly DK, Nguyen T. An effective optimum design for passive viscous damping control using FVDs/VWDs in multi-story buildings. Structures. 2024;67:107004.

[5] Watson WR, Jones MG, Parrott TL, Sobieski J. Assessment of equation solvers and optimization techniques for nonaxisymmetric liners. AIAA J. 2004;42(10):2010–8. doi: 10.2514/1.9020

[6] Beck B, Schiller N, Jones M. Impedance assessment of an acoustic metamaterial-inspired acoustic liner. J Acoust Soc Am. 2013;134:4222.

[7] Groby JP, Huang W, Lardeau A, Auregan Y. The use of slow waves to design simple sound absorbing materials. J Appl Phys. 2015;117:124903.

[8] Fuller CR. Propagation and radiation of sound from flanged circular ducts with circumferentially varying wall admittances, I: Semi-infinite ducts. J Sound Vib. 1984;93:321–40.

[9] Regan B, Eaton J. Modeling the influence of acoustic liner non-uniformities on duct modes. J Sound Vib. 1999;219:859–79.

[10] Campos LMBC, Oliveira JMGS. On the acoustic modes in a cylindrical duct with an arbitrary wall impedance distribution. J Acoust Soc Am. 2004;116(6):3336–47. doi: 10.1121/1.1812308 15658686

[11] Afzal M, Nawaz R, Ayub M, Wahab A. Acoustic scattering in flexible waveguide involving step discontinuity. PLoS One. 2014;9(8):e103807. doi: 10.1371/journal.pone.0103807 25084019 PMC4118954

[12] Rawlins AD. Radiation of sound from an unflanged rigid cylindrical duct with an acoustically absorbing internal surface. Proc R Soc Lond. 1978;A361:65–91.

[13] Demir O, Cinar Y. Propagation of sound in an infinite two-part duct carrying mean flow inserted axially into a larger infinite duct with wall impedance discontinuity. J Appl Math Mech. 2009;89:454–65.

[14] Demir A, Buyukaksoy A. Transmission of sound waves in a cylindrical duct with an acoustically lined muffler. Int J Eng Sci. 2003;41:2411–27.

[15] Rawlins D. Wave propagation in a bifurcated impedance-lined cylindrical waveguide. J Eng Math. 2007;59:419–35.

[16] Hassan M. Wave scattering by soft-hard three spaced waveguide. Appl Math Model. 2014;38:4528–37.

[17] Hassan M, Meylan MH, Bashir A. Mode matching analysis for wave scattering in triple and pentafurcated spaced ducts. Math Meth Appl Sci. 2016;39:3043–57.

[18] Nawaz T, Afzal M, Nawaz R. The scattering analysis of trifurcated waveguide involving structural discontinuities. Adv Mech Eng. 2019;11(7):1–10.

[19] Satti JU, Afzal M, Nawaz R. Scattering analysis of a partitioned wave-bearing cavity containing different material properties. Phys Scr. 2019;94(11):5223.

[20] Ayub M, Tiwana MH, Mann AB. Propagation of sound in a duct with mean flow. Commun Nonlin Sci Numer Simul. 2009;14:3578–3590.

[21] Ayub M, Tiwana MH, Mann AB. Acoustic diffraction in a trifurcated waveguide with mean flow. Commun Nonlin Sci Numer Simul. 2010;15:3939–49.

[22] Hassan M, Mahvish N, Nawaz R. Reflected field analysis of soft-hard pentafurcated waveguide. Adv Mech Eng. 2017;9:1–11.

[23] Bi W, Pagneux V, Lafarge D, Auregan Y. Modelling of sound propagation in a non-uniform lined duct using a multi-modal propagation method. J Sound Vib. 2006;289(3):1091–111.

[24] Campos LMBC, Oliveira JMGS. On the acoustic modes in a cylindrical duct with an arbitrary wall impedance distribution. J Acoust Soc Am. 2004;116(6):3336–47. doi: 10.1121/1.1812308 15658686

[25] Bi W, Pagneux V, Lafarge D, Aurégan Y. An improved multimodal method for sound propagation in nonuniform lined ducts. J Acoust Soc Am. 2007;122(1):280–90. doi: 10.1121/1.2736785 17614488

[26] Hein S, Koch W, Nannen L. Trapped modes and Fano resonances in two-dimensional acoustical duct–cavity systems. J Fluid Mech. 2012;692:257–87. doi: 10.1017/jfm.2011.509

[27] Shafique S, Afzal M, Nawaz R. On the attenuation of fluid-structure coupled modes in a non-planar waveguide. Math Mech Solids. 2020;25(10):1–20.

[28] Afzal M, Shafique S. Attenuation analysis of flexural modes with absorbent lined flanges and different edge conditions. J Acoust Soc Am. 2020;148(1):85. doi: 10.1121/10.0001495 32752741

[29] Afzal M, Shafique S, Wahab A. Analysis of traveling waveform of flexible waveguides containing absorbent material along flanged junctions. Commun Non-Linear Sci Numer Simul. 2021;97:105737.

[30] Bilal H, Afzal M. On the extension of the mode-matching procedure for modeling a wave-bearing cavity. Math Mech Solids. 2021. doi: 10.1177/10812865211018746

[31] Afzal M, Satti JU. The traveling wave formulation of a splitting chamber containing reactive components. Arch Appl Mech. 2021;91:1959–80.

[32] Nawaz R, Lawrie JB. Scattering of a fluid-structure coupled wave at a flanged junction between two flexible waveguides. J Acoust Soc Am. 2013;134(3):1939–49. doi: 10.1121/1.4817891 23967927

[33] Shafique S, Afzal M, Nawaz R. On mode matching analysis of fluid-structure coupled wave scattering between two flexible waveguides. Can J Phys. 2017;95(6):581–9.

[34] Peaks N, Abrahams ID. Sound radiation from a semi-infinite lined duct. Wave Motion. 2020;92:102407.

[35] Afzal M, Nawaz T, Nawaz R. Scattering characteristics of planar trifurcated waveguide structure containing multiple discontinuities. Waves Random Complex Media. 2021. doi: 10.1080/17455030.2020.1864062

[36] Nawaz R, Yaseen A, Alahmadi H, Tiryakioglu B. A mode-matching analysis of flexible shells and waveguides with partitioning and muffler conditions. Int J Mech Mater Des. 2024;20(5):1009–28. doi: 10.1007/s10999-024-09710-y

[37] Nawaz R, Yaseen A, Alkinidri MO. Fluid–structure coupled response of dynamical surfaces tailored in a flexible shell. Math Mech Solids. 2023;28(11):2404–19. doi: 10.1177/10812865231166149

[38] Soumini D, Bhaumik B, De S. Combined effect of induced magnetic field and thermal radiation on ternary hybrid nanofluid flow through an inclined catheterized artery with multiple stenosis. Chem Phys Lett. 2023;811:140209.

[39] Dolui S, Bivas B, De S, Changdar S. Effect of a variable magnetic field on peristaltic slip flow of blood-based hybrid nanofluid through a nonuniform annular channel. J Mech Med Biol. 2023;23(1):2250070.

[40] Dolui S, Bivas B, De S, Changdar S. Biomedical simulations of hybrid nano fluid flow through a balloon catheterized stenotic artery with the effects of an inclined magnetic field and variable thermal conductivity. Chem Phys Lett. 2023;829:140756.

[41] Dolui S, Bhaumik B, De S, Changdar S. Nanoparticle aggregation and electro-osmotic propulsion in peristaltic transport of third-grade nanofluids through porous tube. Comput Biol Med. 2024;176:108617. doi: 10.1016/j.compbiomed.2024.108617 38772055

[42] Alahmadi HN, Nawaz R, Alkinidri M. Noise control from dual air cavity membranes in a rigid waveguide. Meccanica. 2022;57:3023–32.

[43] Alahmadi H, Afsar H, Nawaz R, Alkinidri MO. Scattering characteristics through multiple regions of the wavebearing trifurcated waveguide. Waves Random Complex Media. 2022; doi: 10.1080/17455030.2022.2141912

[44] Gabard G, Astley R. A computational mode-matching approach for sound propagation in three-dimensional ducts with flow. J Sound Vib. 2008;314:1103–24.

[45] Lawrie J. On eigenfunction expansions associated with wave propagation along ducts with wave-bearing boundaries. IMA J Appl Math. 2007;72:376–94.

